# Thermoelectric transport with band non-parabolicity

**DOI:** 10.1093/nsr/nwaf434

**Published:** 2025-10-13

**Authors:** Wenqing Zhang

**Affiliations:** Department of Materials Science and Engineering, Southern University of Science and Technology, China

**Figure 1.**
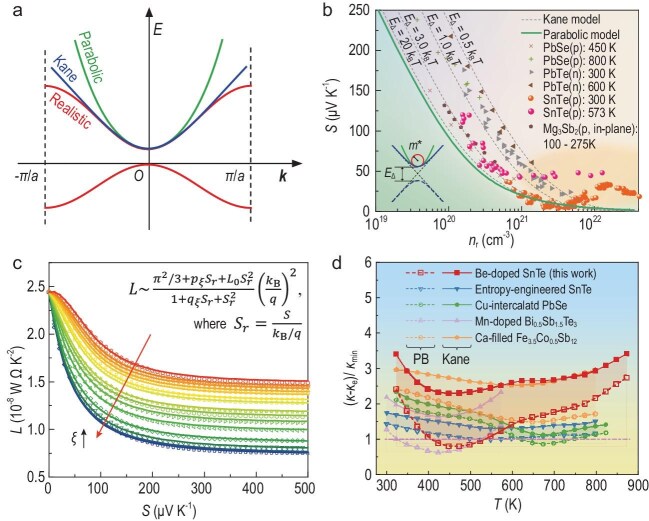
Band non-parabolicity effects on thermoelectric transport properties. (a) Schematic illustration comparing ideal parabolic bands, Kane bands and realistic band structures. (b) Non-parabolicity-induced deviations from classical predictions in the reduced Pisarenko relation. (c) Relationship between the Seebeck coefficient and the Lorenz number with non-parabolicity factor corrections. (d) Prediction of lattice thermal conductivity in parabolic and non-parabolic bands for typical thermoelectrics. The Cahill limit (*κ*_min_) is also plotted for comparison. Reproduced with permission from [4].

Despite the feasibility of obtaining accurate band structures in density-functional computations and angle-resolved photoemission spectroscopy experiments, the classical parabolic band (PB) approximation remains dominant in both theoretical and experimental studies due to its simplicity and analytical tractability. The PB approach simply describes the carrier dispersion near the band extrema with a quadratic energy–momentum relation, $E=\hbar^2k^2/2m^*$, where m* represents the effective mass (Fig. 1a). In thermoelectrics, the PB model serves as a foundational framework, although it is known to be unreliable for complex structures, for analysing transport properties. Key applications include estimating carrier effective mass by using the Pisarenko relation [1] and determining the maximum power factor at an optimal doping concentration through the electronic quality factor [2]. The PB model could also be applied for separating thermal conductivity into the electronic and lattice contributions by using the relationship between the Lorenz numbers (*L*) and the Seebeck coefficients (*S*) and sometimes estimating the potential extra scattering effects from multiple and non-parabolic bands [3].

In a recent work published in *National Science Review*, Zhu and co-workers re-examined the predictions of the classical PB model against density functional theory calculations across various classical semiconductors [4]. Surprisingly, even for certain lightly doped systems in which the PB model is expected to work well, Zhu *et al.* found strong deviation of transport properties from the PB-based Pisarenko relation

and the *L*–*S* relation. By analysing the transport in full bands in Boltzmann transport theory, the authors revealed that *L* and *S* possess distinct non-parabolicity-induced non-local features in the energy dimension. Consequently, the carrier transport behaviour remains profoundly influenced by the linear dispersion regions deep within the conduction or valence bands (Fig. 1b), well beyond the classical Fermi window (a few k_B_T). This finding underscores

the necessity for incorporating band non-parabolicity when analysing transport properties in thermoelectric materials.

Interestingly, the study also found that a simple parameter—the non-parabolicity factor *ξ*—could be defined to accurately quantify the impact of band non-parabolicity on thermoelectric transport properties. The *ξ* defined by referring to Seebeck under the non-degenerate limit transits from 0 to 1 with the evolution of the band from the quadratic PB to the non-parabolic linear band. The study proves that the optimal power factor decreases proportionally to (1 – *ξ*)^2^, while *L* declines as 2 – 2*ξ* + *ξ*^2^ for non-degenerate semiconductors. An analytical *ξ*-corrected *L*–*S* relation (Fig. 1c) could also be established to enable the rapid determination of accurate *L* at practical doping concentrations, facilitating reliable estimations of electronic and lattice thermal conductivities. The study shows that, in several classic thermoelectric materials including SnTe, CoSb_3_ and Bi_2_Te_3_, the lattice thermal conductivity estimated by using the PB model can be unphysically low—even falling below the Cahill thermal conductivity limit [5]. By incorporating *ξ*-based corrections, these underestimations can be effectively corrected (Fig. 1d).

In summary, the band non-parabolicity and the factor *ξ* provide an easy and robust framework for refining the classical PB model and enhance the reliability of thermoelectric property evaluations. They may also facilitate innovations in optimizing material performance across various semiconductor applications.
